# Simple allele-discriminating PCR for cost-effective and rapid genotyping and mapping

**DOI:** 10.1186/1746-4811-5-1

**Published:** 2009-01-08

**Authors:** Minh Bui, Zhongchi Liu

**Affiliations:** 1 Department of Cell Biology and Molecular Genetics, University of Maryland, College Park, Maryland 20742, USA; 2 Department of Biology Graduate Program, University of Maryland, College Park, Maryland 20742, USA

## Abstract

**Background:**

Single nucleotide polymorphisms (SNPs) are widely observed between individuals, ecotypes, and species, serving as an invaluable molecular marker for genetic, genomic, ecological and evolutionary studies. Although, a large number of SNP-discriminating methods are currently available, few are suited for low-throughput and low-cost applications. Here, we describe a genotyping method named Simple Allele-discriminating PCR (SAP), which is ideally suited for the small-scale genotyping and gene mapping routinely performed in small to medium research or teaching laboratories.

**Results:**

We demonstrate the feasibility and application of SAP to discriminate wild type alleles from their respective mutant alleles in *Arabidopsis thaliana*. Although the design principle was previously described, it is unclear if the method is technically robust, reliable, and applicable. Three primers were designed for each individual SNP or allele with two allele-discriminating forward primers (one for wild type and one for the mutant allele) and a common reverse primer. The two allele-discriminating forward primers are designed so that each incorporates one additional mismatch at the adjacent (penultimate) site from the SNP, resulting in two mismatches between the primer and its non-target template and one mismatch between the primer and its target template. The presence or absence of the wild type or the mutant allele correlates with the presence or absence of respective PCR product. The presence of both wild type-specific and mutant-specific PCR products would indicate heterozygosity. SAP is shown here to discriminate three mutant alleles (*lug-3*, *lug-16*, and *luh-1*) from their respective wild type alleles. In addition, the SAP principle is shown to work in conjunction with fluorophore-labeled primers, demonstrating the feasibility of applying SAP to high throughput SNP analyses.

**Conclusion:**

SAP offers an excellent alternative to existing SNP-discrimination methods such as Cleaved Amplified Polymorphic Sequence (CAPS) or derived CAPS (dCAPS). It can also be adapted for high throughput SNP analyses by incorporating fluorophore-labeled primers. SAP is reliable, cost-effective, fast, and simple, and can be applied to all organisms not limited to *Arabidopsis thaliana*.

## Background

Genetic and genomic research has entered a new era with the ever-improving and novel sequencing technologies [1]. Researchers, now more than ever, are taking advantage of the available genomic information for research, teaching, and applications. Single Nucleotide Polymorphism (SNP), the most abundant form of DNA polymorphisms, serves as the most valuable molecular marker for research and application, including the detection of risk associated alleles linked to human diseases [2], the study of evolutionary conservations between different species [3], gene mapping and cloning [4], and crop breeding [5]. In many small to medium size academic laboratories as well as teaching laboratories around the world that utilize *Arabidopsis thaliana *or other genetic model systems, SNPs have become indispensable for genotyping progeny of genetic crosses, discriminating between mutant alleles from wild type alleles or isolating genes using the map-based approach. Efficient and robust genotyping assays are also essential for the identification of individuals carrying suppressor or enhancer mutations that manifest no visible phenotypes of their own [6]. Therefore, robust, reliable, inexpensive, and fast SNP-discriminating methods are needed.

Currently, a large variety of techniques for high-throughput SNP genotyping are available [7,8]. They can be grouped into four main classes: allele-specific hybridization, allele-specific nucleotide incorporation, allele-specific oligonuleotide ligation, and allele-specific invasive cleavage. For example, the TaqMan genotyping method [9] and the Amplifluor SNP HT genotyping System [10] are PCR-based, suitable for large scale high-throughput applications. Both methods, however, require expensive instrumentation and reagents such as synthetic oligonucleotides labeled with different fluorescent dyes. Various genome re-sequencing methods are also extremely powerful for large scale SNP-discrimination [11,12], yet are impractical for assaying a selected set of SNPs in specific genomic regions, and are usually beyond the reach of small to medium sized laboratories with limited resources.

To date, a widely utilized SNP detection method for low-throughput applications in plant research is the Cleaved Amplified Polymorphic Sequence (CAPS), which requires locus-or gene-specific primers to amplify the region of interest, followed by restriction enzyme digestion, and electrophoresis [13]. In a modified CAPS method called "derived CAPS" (dCAPS) [14], an engineered primer creates a restriction enzyme recognition site that can be used to distinguish the targeted SNP. dCAPS is more widely applicable than CAPS because it does not require the SNP to create or destroy a restriction enzyme site. While CAPS and dCAPS are suitable for small to medium scale genotyping, both methods require enzymatic digestion, increasing the cost as well as experimental time. One serious limitation of CAPS and dCAPS is that the restriction enzyme required could be inefficient and costly and that incomplete enzyme digestion hinders one's ability to distinguish heterozygocity from homozygocity of the tested SNP.

During the course of genetic research in construction of double mutants between *leunig (lug) *and *leunig-homolog *(*luh*) [15], we encountered situations in which the dCAPS markers for *lug *and *luh *mutations yielded ambiguous results. We first turned to direct sequencing and subsequently to the Amplifluor SNP HT genotyping System [10]. These methods, while reliable, tend to have a high cost when the number of mutants requiring genotyping increases. In addition, Amplifluor SNP HT requires the access to a real time PCR machine not readily available to us. We searched for alternative genotyping methods and came across with the "amplification refractory mutation system (ARMS)" [16] developed more than 10 years ago in mammalian systems.

The ARMS technique is based on the extension of primer only when its 3'-end is a perfect complement to the allele present in the input sample. However, when terminal mismatching has only weak-destabilizing effect, single mismatch at the terminal base may not discriminate between wild type and mutant templates. Therefore, an additional deliberate mismatch is introduced at the penultimate (second to the terminal) base of the primer to increase the specificity of the PCR reaction. As different mismatches have different destabilizing effects [16], both the terminal and the penultimate mismatches are considered together. If the terminal and natural mismatch is highly unstable, a weak additional mismatch will be introduced at the penultimate site, and vice versa. This principle is further elaborated recently in a graphic dial [17] and can now be designed through a website .

Based on the principle of ARMS, we designed allele-specific primers by introducing an additional mismatch at the penultimate site aimed at destabilizing base pairing between the primers and corresponding non-target templates. We demonstrate that this method offers an excellent alternative to CAPS or dCAPS because of its simplicity, low cost, robustness, speed, and reliability. We named this method SAP (Simple Allele-discriminating PCR) instead of ARMS (amplified refractory mutation system) as SAP more readily explains its application and thus may help popularize its utility. We describe primer design rules and show the successful application of the SAP principle to fluorescent-labeled universal primers in allele-discrimination PCR, allowing high throughput applications. The SAP method provides a practical and useful alternative to existing genotyping methods and will greatly facilitate plant research and teaching.

## Results

### SAP primer design for genotyping three mutant alleles in *Arabidopsis thaliana*

To discriminate single base changes between wild type and the mutant allele, a forward primer that exclusively anneals to WT and another forward primer that exclusively anneals to the mutant allele are designed. These two allele-specific (AS) primers are paired with a common reverse primer for standard PCR reactions. The AS primers are designed based on the principle that if the existing SNP mismatch results in a weak destabilization between the AS primer and its non-template target, a strong destabilizing mismatch will be introduced at the penultimate site. Conversely, if the SNP mismatch already has a strong destabilizing effect, a weak destabilizing mismatch should be introduced at the penultimate site. If a medium destabilizing effect exists at the SNP mismatch, a weak or medium mismatch will be created at the penultimate site.

Table 1 indicates the weak, medium, strong, or maximum destabilization effect of each mismatched pair, based on Little (1995) [16]. In general, the purine-pyrimidine mispairing (G-T and A-C) are more stable and exhibit a weaker destabilization effect than the purine-purine or pyrimidine-pyrimidine mismatches as purine-pyrimidine mismatches still form two hydrogen bonds in a geometry similar to G-C and A-T, and they do not require contracting or expanding the double helix. Pyrimidine-pyrimidine or purine-purine mispairings, in contrast, are more unstable because of the altered geometry in the double helix as well as reduced hydrogen bonding. For more detailed analyses of thermodynamics of mismatches, one can consult Peyret et al. (1999) [18].

**Table 1:** The strength of destabilization for all combinations of nucleotide pairing

**Base Pairing**	**Destabilization Strength**
GA, CT, TT	Maximum
CC	Strong
AA, GG	Medium
CA, GT	Weak
AT, GC	None

**Figure 1:**
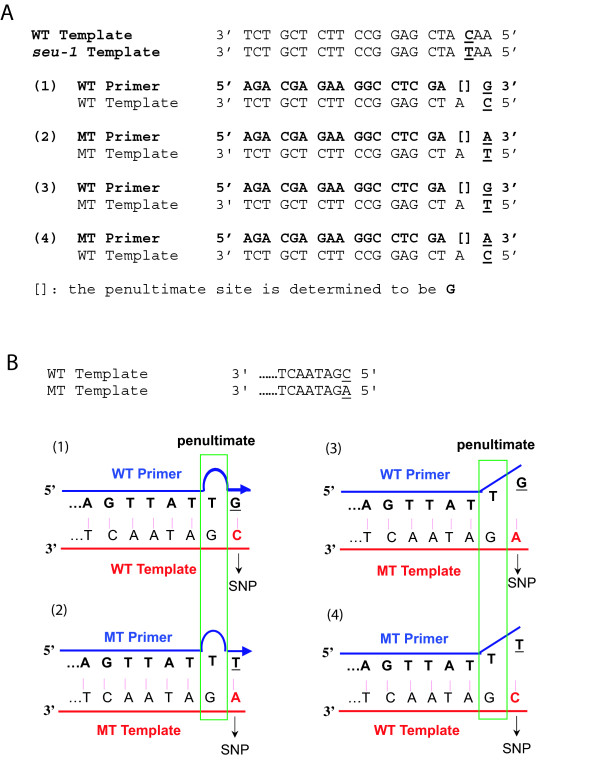
**Illustration of the SAP principle**. (A) A step-by-step illustration of the AS primer design for the *Arabidopsis seu-1 *mutant. The WT (SEU) sequence and the *seu-1 *mutant sequence are shown on top. The mutated base is underlined. The WT (SEU)-specific primer is first designed based on its complementarity to WT template sequence shown in (1); the MT (*seu-1*)-specific primer sequence is designed based on its complementarity to the MT template sequence shown in (2). The primer sequence is always from 5' (left) to 3' (right). The penultimate base in the AS primers is indicated by a bracket. Subsequently, the WT primer is paired against the MT template (3) to determine the terminal mismatch (GT). Similarly, MT primer sequence is paired against WT template sequence (4) to determine the terminal mismatch (AC). By referring to Table 1, the GT and AC terminal mismatches identified above both exhibit weak destabilization effect. Thus, the penultimate mismatch should exhibit a strong destabilization. By referring to Table 1, the strongest destabilization mismatch that involves "A" is "GA". Therefore, G is chosen at the penultimate site of both WT and MT AS primers. (B) Four possible annealing scenarios for a hypothetical C to A mutation, which is underlined. Because the terminal mismatches (GA and TC) are strong destabilizing, the penultimate site thus selects a weak destabilizing mismatch (TG), which is indicated within the green rectangle. (1) Proper annealing of a WT primer to the WT template, which will lead to successful PCR amplification. (2) Stable annealing of the MT primer to the MT template, leading to successful PCR amplification. (3) Unstable pairing of the WT primer to the MT template due to two consecutive mismatches. No PCR product is expected. (4) Unstable pairing of the MT primer to the WT template. No PCR amplification is expected.

When designing the AS primers, the specific type of nucleotide introduced at the penultimate site should be determined by consulting Table 1. A step-by-step illustration of AS primer design for the *seuss (seu)-1 *mutant [19] is shown in Fig. 1A. The terminal mismatches (GT, AC) in this case are weak destabilizing, thus a strong destabilizing mismatch (GA) is introduced at the penultimate site. A second example (Fig. 1B) shows a C (WT) to A (MT) mutation, which resulted in strong destabilizing mismatches at the terminal site between the WT and MT primers and their corresponding non-target templates, respectively. As a result, a weak destabilizing mismatch is introduced at the penultimate site. A web-based computational design tool using this principle can be found at [17].

**Figure 2:**
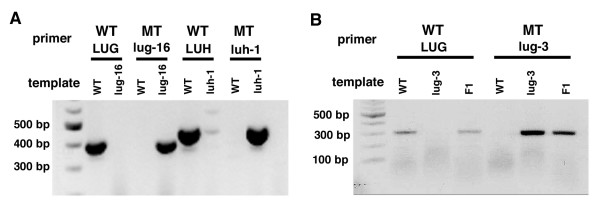
**SAP-based genotyping of three different mutant alleles**. (A) WT LUG and MT *lug-16 *genotypes were identified by the positive amplification of a 401 bp band when the WT (LUG) primer and the *lug-16 *MT primer amplify their WT and MT target template DNA, respectively. Similarly, WT (LUH) and MT *luh-1 *genotypes were identified when a 456 bp PCR fragment was amplified with respective primers. (B) Presence of WT (LUG) and MT *lug-3 *template DNA correlates with the amplification of a 301 bp PCR band using respective WT (LUG) and MT *lug-3 *primers. A heterozygote (F1 progeny of a cross between wild type and *lug-3*) correlates with the positive PCR amplifications with both WT (LUG) or MT *lug-3 *primers.

The initial application of the SAP assay to genotyping three mutant alleles, *lug-16, luh-1 *and *lug-3*, is shown (Fig. 2A, B; Table 2). Subsequently, several other mutations were genotyped by the SAP (data not shown). In all cases, the SAP assay was successful. For example, Fig. 2A shows the PCR amplification of WT template with the WT (LUG) primer and the amplification of *lug-16 *MT template by the *lug-16 *MT primer. It also shows the failure of PCR amplification of WT template by the *lug-16 *MT primer, and failure of PCR amplification of *lug-16 *MT template by the WT (LUG) primer, suggesting that the WT (LUG) and MT (*lug-16*) primers are highly specific to their target templates. Similar genotyping result was obtained for *luh-1 *(Fig. 2A). In addition, Fig. 2B illustrates the utility of SAP in identifying an F1 progeny (heterozygote) of a genetic cross between wild type and *lug-3 *mutants.

**Table 2:** Primer sequences for three different alleles

**SNP**	**Primer**	**Direction**	**Sequence (5'-3')**
**lug-16**	WT-Specific	Reverse	CCACCAGGTGCGTCAATATC
	
	Mutant-Specific	Reverse	CCACCAGGTGCGTCAATATT
	
	Common	Forward	TTGTATGCAAGTATGTGACTTTA

lug-16* (Amplifluor)	WT-FAM	Reverse	GAAGGTGACCAAGTTCATGCTTCCACCAGGTGCGTCAATATC
	
	Mutant-JOE	Reverse	GAAGGTCGGAGTCAACGGATTTCCACCAGGTGCGTCAATATT
	
	Common HT	Forward	CTGCAGTTGCTCTGTTTCCTAA

**luh-1**	WT-Specific	Forward	GGAGGGTTTCTTTTTGAGTTG
	
	Mutant-Specific	Forward	TGGAGGGTTTCTTTTTGAGTTA
	
	Common	Reverse	CCATGATGGTTTGTTGCTGAT

**lug-3**	WT-Specific	Reverse	TTGATGTTGTTGTTGCTGCGG
	
	Mutant-Specific	Reverse	TTGATGTTGTTGTTGCTGCCA
	
	Common	Forward	ACTAAGCTGGAGTATTTCTATTT

*: The underlined sequences are 5' extended primer sequences that specifically pair with the 3' region of the FAM or JOE universal fluorescent primers, respectively of the Amplifluor SNPs Genotyping System.

The SAP assay is normally set up in two parallel PCR reactions. One set of PCR reaction combines the WT-specific primer with the common reverse primer. The second set of PCR reaction combines the MT-specific primer with the same common reverse primer. When the SAP assay is first developed for a specific SNP, different annealing temperatures should be tested using WT and MT DNA templates to identify the optimal annealing temperature that allows positive amplification of AS primers with respective target templates and negative amplification with non-target templates. Ideally, the optimal anneal temperature for the WT-specific amplification is the same as that of the MT-specific amplification, allowing for single PCR runs. However, this is sometimes difficult to achieve, and separate PCR runs using different annealing temperatures for WT and MT-specific PCR reactions are necessary.

### Feasibility in high-throughput applications

**Figure 3:**
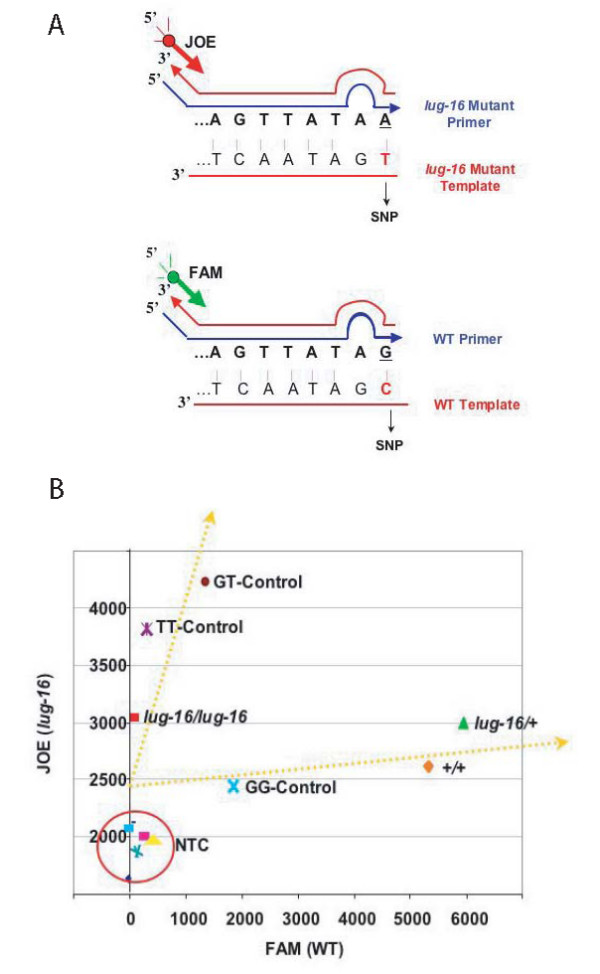
**Adaptation of SAP for high throughput applications**. (A) Diagram illustrating the Amplifluor SNP genotyping assay system. The allele-specific primer each has a unique 5' tail sequence that is identical to the 3' region of one of the Amplifluor SNP Universal Primers (FAM or JOE indicated by green and red arrows respectively). When combined with the common reverse primer, PCR amplification results in the synthesis of the tail sequence complement (thin red line). The Amplifluor^® ^SNPs Universal Primer then anneals specifically to the tail of reverse complement and is elongated by Taq Polymerase. Subsequent PCR cycles unfold the hairpin structure (indicated by filled circle) of the Amplifluor^® ^SNPs Universal Primers, which results in fluorescent signals. (B) A scatter plot showing results of a SAP-based Amplifluor SNP assay. X-axis represents the FAM signal measuring the amplification of WT LUG (+), and the Y-axis indicates the JOE signal that measures *lug-16*-specific PCR amplification. Two types of controls were used. First, the manufacturer's template controls (GG, GT, TT) utilize FAM/JOE SNP primers and the control templates (GG, TT, and GT), both of which are provided by the manufacturer's kit. Second, the non-target control (NTC) uses water instead of DNA template. Results of three experimental samples (*lug-16/lug-16*, *lug-16/+*, and +/+) are shown. DNA template was from known genotype. The experiment has been performed twice with similar results. The result from one such an experiment is shown.

In certain instances, when large-scale analyses are required or when there is a small amount of genetic material, SAP can be applied in a high-throughput and highly sensitive manner. To demonstrate such an application, the AS primer design principle was utilized and adapted to the Amplifluor SNPs Genotyping System (Chemicon) [10]. This technology uses energy transfer (ET) universal primers that generate fluorescent PCR products (Fig. 3A). While the allele-discriminating principle is the same as SAP, the detection of the PCR products requires a machine capable of reading fluorescence such as a fluorescenct plate reader or a qRT-PCR machine.

In our experiment, the WT primer is annealed to the Amplifluor SNPs Genotyping primer FAM, while the MT *lug-16 *primer is annealed to the Amplifluor SNPs Genotyping primer JOE. After PCR, the data was transferred to Microsoft Excel, and scatter plots were generated (Fig. 3B). Homozygous wild type (+/+) showed a high FAM signal and some background JOE signal. In contrast, homozygous mutant (*lug-16/lug-16*) showed a high level of JOE signal and some background FAM signal. Heterozygote (*lug-16/+) *showed significant signal from both JOE and FAM. The successful discrimination between *lug-16/lug-16 *homozygotes, wild type (+/+), and *lug-16/+ *heterozygotes indicates that the SAP-based principle can be applied to high-throughput and highly sensitive applications. In addition, this method is highly sensitive, requiring only 0.4 ng template DNA in 10 microliter PCR reactions.

Unlike the low throughput examples discussed earlier, both the WT and the MT AS primers are added into the same PCR mix and used to amplify their target templates using the same PCR program. If the two AS primers do not amplify their target DNA with equal efficiency, one fluorescent signal (such as FAM shown in Fig. 3B) could be significantly higher than that of the other fluorescent signal (such as JOE in Fig. 3B). Therefore, it is important to always include wild type and mutant control templates in the same experiment.

## Methods

### Plant growth and DNA extraction

*Arabidopsis thaliana *wild type and mutant plants were grown under 16-hour long day conditions at 20°C and 65% humidity for 4 weeks. One to two leaves were collected from individual *Arabidopsis *plants, and DNA was extracted using Edwards buffer (200 mM Tris, pH: 7.5; 250 mM NaCl; 25 mM EDTA, pH: 8.0; 0.5% SDS), precipitated with isopropanol, washed with 70% ethanol, and resuspended in 50 to 100 μL distilled water, 2 μL of which (roughly about 10 ng genomic DNA) was used in 20 μL PCR reactions.

DNA template was sometimes obtained through the FTA card (Whatman) following manufacturer's instructions. One single leaf was pressed onto the FTA card and allowed to dry. 1.2 mm diameter discs were punched out of the DNA-containing FTA cards using the 1.2 mm micro punch. The discs were first washed with 20 μL FTA Purification Reagent (Whatman) and washed again with 20 μL 1× TE buffer. Each DNA disc was used directly in individual PCR reactions.

### Primers and PCR

Primers were designed as described in the Result section. PCR program for all alleles described here was the same, beginning with 94°C for 3 minutes, followed by 35 cycles of 94°C for 20 seconds, 55–57°C for 20 seconds, and 72°C for 40 seconds, and ended by 72°C for 3 minutes. WT and MT primer pairs were designed to have similar annealing temperatures to allow simultaneous PCR. Primer sequences are provided in Table 2. Standard PCR reaction was used with the final primer concentration at 0.5 μM and the final dNTP concentration at 0.2 μM in a 20 μL PCR reaction. Taq DNA Polymerase was purchased from GeneScript Corporation (Cat# E00007). 1% agarose gels were made with Invitrogen's UltraPure Agarose. 5 μL PCR reaction was loaded in each lane of the 1% agarose gel.

### High-throughput application

The Amplifluor SNPs Genotyping System for Assay Development kit was purchased from Chemicon International (Millipore Cat# S7907). AS primers for WT LUG and MT *lug-16 *were designed with a 5' tail sequence identical to the 3' region of the FAM or JOE universal primers, respectively (Table 2). PCR reaction mixture and PCR program were set up following the manufacturer's instruction and using the Platinum Taq DNA Polymerase (Invitrogen). End-point fluorescence detection was carried out using BioRad's iQ5 Multicolor Real-Time PCR Detection System and software.

## Discussion

We describe a simple SNP-discriminating method and demonstrate its utility for plant research. Although the design principle was previously described [16,17], it is unclear if it has been successfully utilized in any organisms, nor is it known if the method is technically robust, reliable, and applicable.

Several important lessons were learned in the course of developing the SAP assay. First, a primer that is too stable will not distinguish between the target and the non-target templates. In contrast, an unstable primer will not effectively amplify its target template. To weaken undesirable stability between the primer and its non-template target, either the primer length is reduced, or the PCR annealing temperature is increased. The general rule of thumb is to maintain primer G/C contents at 36% to 66%, primer length between 18 and 22 bases, the amplicon size around 200–600 bases, and the annealing temperature between 55°C to 60°C. WT and MT allele-specific primers are best kept at similar length to allow for same PCR conditions. When the last nucleotide at the 3' end of the AS-primer is a G or C, there is often an increased likelihood of a faint, non-specific background PCR band. Accordingly, an increase in annealing temperature or a shortening of primer length may be necessary. Finally, PCR conditions have to be first optimized using the wild type and mutant DNA template controls. PCR optimizing runs on a temperature gradient are highly recommended when one develops the SAP assay. It is also possible to further optimize the assay by adjusting appropriate primer and dNTP concentrations.

## Conclusion

The aforementioned SAP method described is a cost-effective, time-efficient, robust and reliable method for the identification and discrimination of different alleles. SAP offers several advantages over existing CAPS and dCAPS genotyping assays and can be adapted for high-throughput applications. SAP may be broadly applied to a wide range of research in any organism.

## Abbreviations

AS: Allele Specific; PCR: Polymerase Chain Reaction; SAP: Simple Allele-discriminating PCR; SNP: Single Nucleotide Polymorphism; WT: Wild Type; MT: Mutant; WASP: Web-based Allele-Specific PCR.

## Competing interests

The authors declare that they have no competing interests.

## Authors' contributions

MB and ZL conceived the project, designed experiments, and prepared the manuscript. MB conducted the experiments. ZL acquired funding and approved the final version of the manuscript.
